# MicroRNA expression in human retinal pigment epithelial (ARPE-19) cells: Increased expression of microRNA-9 by N-(4-Hydroxyphenyl)retinamide

**Published:** 2010-08-04

**Authors:** R. Krishnan Kutty, William Samuel, Cynthia Jaworski, Todd Duncan, Chandrasekharam N. Nagineni, Nalini Raghavachari, Barbara Wiggert, T. Michael Redmond

**Affiliations:** 1 Laboratory of Retinal Cell and Molecular Biology, National Eye Institute, National Institutes of Health, Bethesda, MD; 2 Laboratory of Immunology, National Eye Institute, National Institutes of Health, Bethesda, MD; 3 Genomics Core Facility, Pulmonary and Vascular Medicine Branch, National Heart Lung and Blood Institute, National Institutes of Health, Bethesda, MD

## Abstract

**Purpose:**

MicroRNAs (miRNAs) are important regulators of many cellular functions due to their ability to target mRNAs for degradation or translational inhibition. Previous studies have reported that the expression of microRNA-9 (miR-9) is regulated by retinoic acid and reactive oxygen species (ROS). We have previously shown that *N*-(4-hydoxyphenyl)-retinamide (4HPR), a retinoic acid derivative, induces ROS generation and apoptosis in cultured human retinal pigment epithelial (RPE) cells, known as ARPE-19 cells. The aim of the present study was to investigate the expression of miR-9 in ARPE-19 cells in response to 4HPR treatment, and to identify other miRNAs normally expressed in these cells.

**Methods:**

ARPE-19 cells in culture were treated with 4HPR, the total RNA fractions were isolated, and the expression of various miRNAs and mRNAs was analyzed using real-time PCR. The miRNA expression profile of ARPE-19 cells was analyzed using microarray hybridization.

**Results:**

Treatment of ARPE-19 cells with 4HPR resulted in apoptosis characterized by the increased expression of *HMOX1* and *GADD153* genes. A twofold increase in the expression of miR-9 was also observed during this response. Potential binding sites for the transcription factors encoded by *CEBPA* and *CEBPB* genes were found to be present in the putative promoter regions of all three genes encoding miR-9. 4HPR-induced miR-9 expression was associated with parallel increases in the expression of these transcription factor genes. 5-Aza-2’-deoxycytidine, a methyl transferase inhibitor, also increased the expression of miR-9 in ARPE-19 cells. Microarray hybridization analysis identified let-7b, let-7a, miR-125b, miR-24, miR-320, miR-23b, let-7e, and let-7d as the most abundant miRNAs normally expressed in ARPE-19 cells. These miRNAs are known to regulate cell growth, differentiation or development. The 4HPR treatment increased the expression of miR-16, miR-26b, miR-23a, and miR-15b in ARPE-19 cells, although these increases were modest when compared to the increase in the expression of miR-9.

**Conclusions:**

Our studies demonstrate that miR-9 is expressed in the RPE cell line ARPE-19, and its expression is increased by a retinoic acid derivative and by an inhibitor of promoter hypermethylation. Several miRNAs with inherent ability to regulate cell growth, differentiation and development are also normally expressed in ARPE-19 cells. Thus, miR-9 and other miRNAs could be important in maintaining RPE cell function.

## Introduction

MicroRNA (miRNA) is a class of single-stranded noncoding small (~22 nucleotides) RNA molecules known to regulate gene expression posttranscriptionally [1,2]. The miRNAs, encoded by genes localized to various chromosomes, are initially transcribed as primary transcripts (pri-miRNAs), then converted to pre-miRNAs and subsequently processed to mature miRNAs, which are essential components of the RNA-initiated silencing complex (RISC). An miRNA can function as a posttranscriptional silencer of gene expression either by destabilizing its target transcripts or by inhibiting their translation. A perfect complementarity between the miRNA and its target mRNA often results in the rapid degradation of the latter. A single miRNA can bind to the 3′-untranslated region (3′UTR) of many target gene transcripts and thereby inhibit their translation. The translational repression requires only a partial complementarity between the miRNA and its target mRNAs [2]. Recent studies have identified miR-9 as one such miRNA with an important role in cell growth, differentiation, neurogenesis, immunity and oncogenesis due to its ability to target translational inhibition of many genes, including *ONECUT2* (one cut homeobox 2), *REST* (RE1-silencing transcription factor), *TLX* (*NR2E1*, nuclear receptor subfamily 2, group E, member 1), and *NFKB1* (nuclear factor of kappa light polypeptide gene enhancer in B-cells) [3-9]. The expression of miR-9 is generally suppressed in cases of cancer due to hypermethylation of the promoter regions of genes encoding it [10-13]. The expression of miR-9 is altered in brains affected by Alzheimer disease, and BACE1/beta-secretase is a target for translational inhibition by this microRNA [14,15]. Decreased expression of miR-9 is observed in presenilin-1 null mice [16]. Its expression is also reported to be regulated by retinoic acid, oxidative stress, alcohol and pro-inflammatory agents [7,9,17-19].

A normally functioning retinal pigment epithelium (RPE) is indispensible for vision, and impaired function as a result of oxidative stress is thought to be a major factor responsible for the development of retinal degenerative diseases, such as age-related macular degeneration [20]. ARPE-19, a cell line derived from human RPE, has been widely used to investigate the response of RPE to oxidative stress [21-25]. A previous study from our laboratory has shown that the retinoic acid derivative N-(4-hydroxyphenyl) retinamide (4HPR, fenretinide) can generate oxidative stress, increase the expression of *HMOX1* (heme oxygenase [decycling] 1) and *GADD153* (*DDIT3*, DNA-damage-inducible transcript 3) genes and subsequently induce apoptosis in ARPE-19 cells [26]. We reasoned that miR-9 expression could be altered during this process since its expression is reported to be regulated by both retinoic acid and oxidative stress [9,17,18]. Therefore, we investigated the expression of miR-9 in ARPE-19 cells in response to 4HPR-induced *HMOX1* and *GADD153* expression and apoptosis. We then identified other miRNAs normally expressed in ARPE-19 cells using microarray analysis and determined if the expression levels of some of these miRNAs were also affected by 4HPR.

## Methods

### Cell culture

The ARPE-19 human RPE cell line at passage 18 was obtained from ATCC (Manassas, VA) and the experiments were performed using cells from passages 20 through 24. The cells were grown in Dulbecco's modified Eagle's medium containing nutrient mixture F12, 50/50 mix (Cellgro, Herndon, VA) supplemented with 5% fetal bovine serum, 2 mM L-glutamine, 1 mM sodium pyruvate, 0.1 mM non-essential amino acids and penicillin (100 U/ml) and streptomycin (100 μg/ml), as described previously [26]. Cells were seeded onto tissue culture plates at a density of 2×10^5^ cells/ml in complete medium and allowed to grow at 37 °C in a humidified environment of 5% CO_2_ in air to reach about 80% confluence (1–2 days). The culture medium was then replaced with fresh serum-free medium containing penicillin (100 U/ml) and streptomycin (100 μg/ml) before treating the cells with various agents. N-(4-hydroxyphenyl)retinamide (4HPR) was purchased from Biomol (Plymouth Meeting, PA) and dissolved at a concentration of 10 mM in DMSO before being added to the cell culture medium to the required concentration. The controls received an equal volume of vehicle. Treatments were performed under subdued light and other conditions for 24 h, as reported previously [26]. The ARPE-19 cells were treated with 5-aza-2’-deoxycytidine (Sigma-Aldrich, St. Louis, MO). The compound was first dissolved in DMSO before adding it to the cell culture medium to a final concentration of 1 µM. This treatment lasted 3 days, the culture medium being replaced every 24 h with fresh medium containing the compound. Sodium meta-arsenite and menadione were obtained from Sigma-Aldrich, their stock solutions were prepared in water and the cells were treated with these compounds for 24 h.

### Apoptosis ELISA

Detection of apoptosis in ARPE-19 cells was performed by quantitative sandwich-enzyme-immunoassay using mouse monoclonal antibodies directed against DNA and histones (Cell Death Detection Elisa kit; Roche, Indianapolis, IN), as previously described [26]. ARPE-19 cells were grown on 24-well tissue culture plates, treated with 4HPR for 24 h, and lysed by incubating with lysis buffer (250 µl/well) for 30 min at room temperature. The cell lysates were then centrifuged at 250× g for 10 min, and 10 µl aliquots of supernatants were removed and analyzed using ELISA. The absorbance at 405 nm was measured using a Victor^2^ Multilabel Counter (Perkin Elmer, Waltham, MA).

### Microarray analysis

Microarray hybridization analysis was performed using NCode miRNA Expression Profiling Services (Invitrogen, Carlsbad, CA). Total RNA was isolated from untreated ARPE-19 cells using TRIzol Reagent, and its quality analyzed using agarose gel electrophoresis. Small RNA was enriched from total RNA (20 μg) using the PureLink miRNA Isolation Kit. The enriched miRNA fraction (2.4 μg) was Poly(A) tailed, labeled with Alexa Fluor (NCode miRNA Labeling System) and hybridized to NCode Multi-Species miRNA Microarrays for 4 h at 62 °C. Two RNA samples were analyzed using this procedure on different microarrays containing duplicate spots of each miRNA probe. Hybridization and washing of the arrays were performed according to the standard protocol described in NCode miRNA Labeling System Manual. The arrays were scanned on a GenePix 400B Microarray Scanner (Molecular Devices, Sunnyvale, CA) and images were then analyzed using the GenePix software.

### Quantitative real-time RT–PCR analysis

For quantitative real-time RT–PCR analysis of various transcripts, 2 µg of total RNA extracted from ARPE-19 cells with RNeasy Protect Mini Kit (Qiagen, Valencia, CA) was reverse transcribed using the High Capacity cDNA Archive Kit (Applied Biosystems, Foster City, CA). After reverse transcription, 5 µl of cDNA was used as a template for quantitative real-time PCR, which was performed on an Applied Biosystems 7500 Real-Time PCR System using TaqMan Universal PCR Master Mix and other reagents from Applied Biosystems. The manufacturer's default thermal cycling conditions were followed. Each PCR reaction (20 µl) was set up by using validated TaqMan probes (labeled with reporter dye FAM at the 5′ end) and primers specific for each gene (*GADD153*, *HMOX1*, *CEBPA*, *CEBPB*, and *TGM2* with assay identification numbers Hs00358796_g1, Hs00157965_m1, Hs00269972_s1, Hs00270923_s1, and Hs00190278_m1, respectively). A human *GAPDH* (part number: 4352934E) gene was used as the endogenous control. Gene amplification data were analyzed using Applied Biosystems 7500 System Sequence Detection Software version 1.2.3. The results were expressed as n-fold induction in gene expression, calculated using the ΔΔCT method.

For real-time PCR analysis of miRNAs, total RNA fraction containing miRNAs was prepared from ARPE-19 cells using miRNeasy Mini Kit (Qiagen). RT–PCR analysis was performed on an Applied Biosystems 7500 Real-Time PCR System using individual TaqMan MicroRNA Assays (miR-107, miR-125b, miR-15a, miR-16, miR-193b, miR-210, let-7a, let-7c, let-7d, miR-98, miR-9, miR-34a, miR-26b, miR-24, miR-23a, miR-223, miR-15b, miR-128a, miR-204, or miR-224), TaqMan MicroRNA Reverse Transcription Kit and TaqMan Universal PCR Master Mix, No AmpErase UNG (Applied Biosystems). RNU48 was used as the endogenous control and the ΔΔCT method was employed to estimate miRNA expression.

The expression of selected miRNAs in ARPE-19 cells was verified by real-time PCR employing NCode SYBR Green miRNA qRT–PCR Kit and NCode miRNA First-Strand cDNA Synthesis Kit (Invitrogen). Total RNA samples (1 μg) were first treated with DNase I and then subjected to a polyadenylation reaction before first strand cDNA synthesis with SuperScript III and Universal RT Primer (Invitrogen). Each PCR reaction was performed using a forward primer specific for the tested miRNA and the Universal qPCR Primer. The miRNAs were analyzed and their forward primer sequences were determined as: let-7b, TGA GGT AGT AGG TTG TGT GGT T; miR-125b, TCC CTG AGA CCC TAA CTT GTG A; miR-24, TGG CTC AGT TCA GCA GGA ACA G; miR-23b, ATC ACA TTG CCA GGG ATT ACC; let-7e, TGA GGT AGG AGG TTG TAT AGT; miR-210, CTG TGC GTG TGA CAG CGG CTG A; miR-193B, AAC TGG CCC TCA AAG TCC CGC TTT; miR-423–3P, AGC TCG GTC TGA GGC CCC TCA G. For the analysis of statistical significance, Student’s paired *t*-test was used. All values have been expressed as mean±SD, n=4; p<0.05 denotes statistically significant differences. The results shown are representative of three different experiments.

### Search for potential transcription factor binding sites

DNA sequences (1 kb) upstream of the genes transcribing miR-9 or other miRNAs were examined for the presence of potential transcription factor binding sites. The following Web-based software packages were used, each with default parameters: TESS - Transcription Element Search System [27] and the MatInspector program from Genomatix.

## Results

**Figure 1:**
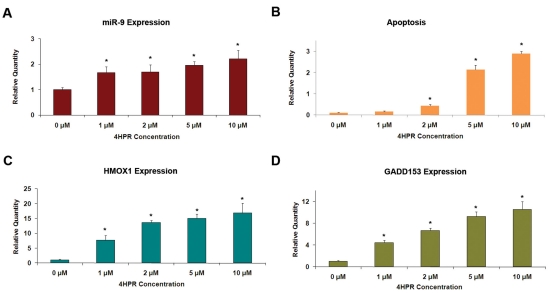
Increased miR-9 expression in ARPE-19 cells during 4HPR-induced apoptosis and the expression of *HMOX1* and *GADD153*. ARPE-19 cells were treated with indicated concentrations of 4HPR for 24 h, and real-time RT–PCR was employed to analyze the expression of miR-9, *HMOX1* and *GADD153*. Formation of mono- and oligonucleosomes was estimated using ELISA as a marker for apoptosis. **A:** 4HPR increased miR-9 expression. **B:** 4HPR-induced apoptosis as indicated by the generation of mono- and oligonucleosomes. **C:** 4HPR-induced *HMOX1* expression. **D:** 4HPR-induced *GADD153* expression. *p<0.05 compared to control, n=4.

We analyzed the expression of miR-9 in ARPE-19 cells in response to 4HPR treatment. The cells were treated with varying concentrations of 4HPR for 24 h, and the miR-9 expression was measured using real-time RT–PCR. An increase in miR-9 expression was observed with increasing concentration of 4HPR (Figure 1). A ~2 fold increase in miR-9 expression was detected when the concentration of 4HPR reached 10 µM. Dimethyl sulfoxide, the vehicle used in this study to dissolve 4HPR, had no effect on the miR-9 expression by itself. ARPE-19 cells from passages 20 to 24 did not show detectable variation in miR-9 expression or its response to 4HPR treatment (data not shown). The 4HPR treatment induced apoptosis, as expected, as indicated by the generation of mono- and oligonucleosomes in the treated cells. We also analyzed the expression of two genes, *HMOX1* (a marker for oxidative stress) and *GADD153*, since we have previously shown that their expression is highly increased during 4HPR-induced apoptosis of ARPE-19 cells [26]. The 4HPR-dependent increase in miR-9 expression paralleled increases in the expression of *HMOX1*and *GADD153*. Thus, treatment of ARPE-19 cells with 4HPR induces expression of miR-9 along with established markers of oxidative stress and apoptosis.

**Figure 2:**
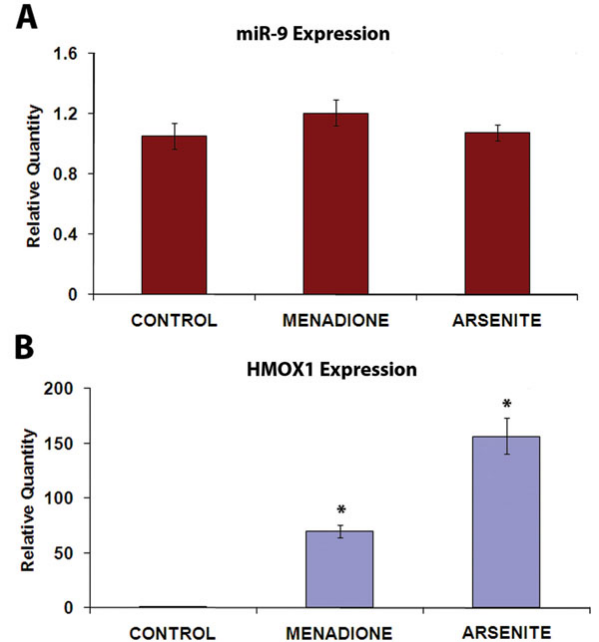
MiR-9 expression in ARPE-19 cells is not affected by oxidative stress. The cells were treated for 24 h with menadione (20 μM) or sodium arsenite (2 μM), agents known to cause oxidative stress. The expression of miR-9 and *HMOX1*, a marker for oxidative stress, was analyzed using real-time RT–PCR. **A:** MiR-9 expression was not affected by menadione and arsenite. **B:** *HMOX1* expression was markedly increased, as expected, by both menadione and arsenite. *p<0.05 compared to control, n=4.

The miR-9 expression in ARPE-19 cells in response to 4HPR treatment was further investigated. Earlier work from our laboratory has shown that 4HPR-induced apoptosis of ARPE-19 cells is preceded by reactive oxygen species (ROS) generation [26]. The increase in *HMOX1* expression is a consequence of oxidative stress. We examined the possibility that the observed increase in the expression of miR-9 in 4HPR-treated cells is mediated by oxidative stress, rather than by 4HPR directly. ARPE-19 cells were treated with menadione and sodium arsenite, agents known to cause oxidative stress, and the miR-9 expression and *HMOX1* expression were then analyzed. Both of these agents induced *HMOX1* expression as expected, but the miR-9 expression was not affected significantly (Figure 2). Thus, oxidative stress per se does not appear to mediate the effect of 4HPR on miR-9 expression.

**Figure 3:**
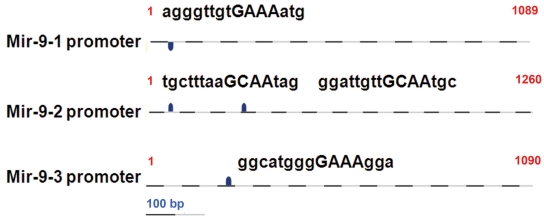
Sequences matching putative binding sites for CEBP transcription factors are present in the 5′-flanking regions of all three human miR-9 genes. DNA sequences (1 kb) upstream of the miR-9 genes were analyzed for the presence of potential transcription factor binding sites, as described in the METHODS.

**Figure 4:**
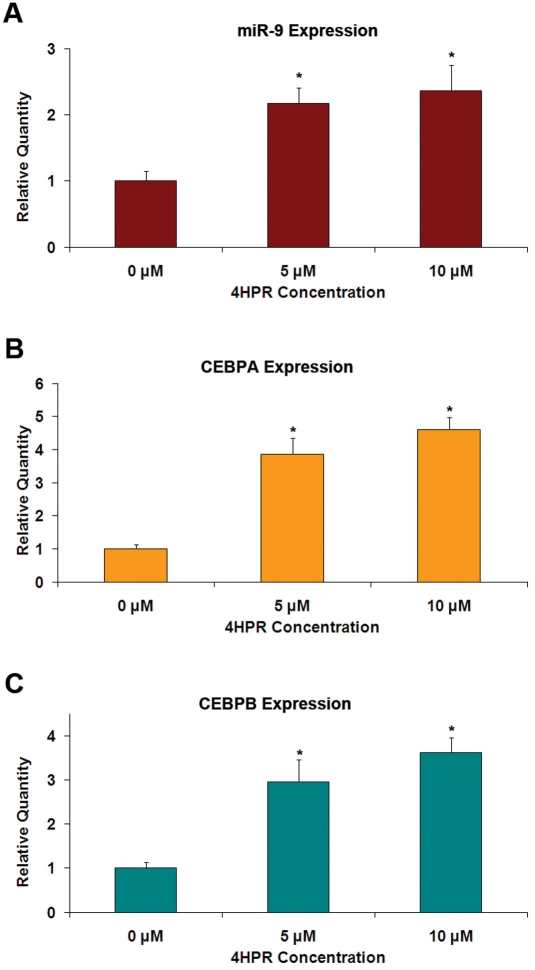
4HPR-induced expression of miR-9 in ARPE-19 cells is associated with increased expression of *CEBPA* and *CEBPB*. The cells were treated with 10 μM of 4HPR for 24 h, and the gene expression was analyzed using real time RT–PCR. **A:** 4HPR increased miR-9 expression. **B:** 4HPR increased *CEBPA* expression. **C:** 4HPR increased *CEBPB* expression. *p<0.05 compared to control, n=4.

We analyzed the putative promoter regions of three genes encoding miR-9 for regulatory elements. As shown in Figure 3, potential binding sites for CCAAT/enhancer binding protein (CEBP) transcription factors, CEBP-α and CEBP-β, are present in the 5′-flanking regions of *MIR9–1*, *MIR9–2*, and *MIR9–3* genes. Thus, it is possible that these transcription factors could regulate the expression of miR-9. As shown in Figure 4, the increase in the expression of miR-9 due to 4HPR is associated with parallel increases in the expression of *CEBPA* and *CEBPB*, the genes encoding CEBP-α and CEBPB-β, respectively.

**Figure 5:**
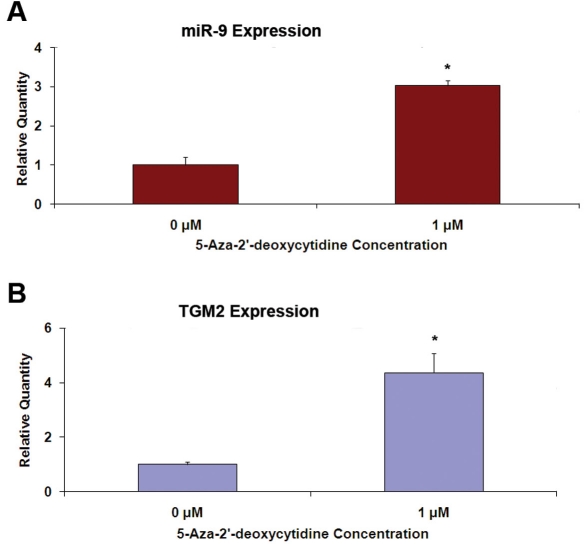
Expression of miR-9 increases in ARPE-19 cells following treatment with 5-aza-2’-deoxycytidine. Cells were treated with 1 μM 5-aza-2’-deoxycytidine for 3 days, and miR-9 expression was estimated using real time RT–PCR. The expression of *TGM2*, a gene known to be upregulated by this treatment, was also analyzed. **A:** 5-aza-2’-deoxycytidine increased miR-9 expression. **B:** 5-aza-2’-deoxycytidine increased *TGM2* expression. *p<0.05 compared to control, n=4.

The expression of miR-9 genes could be suppressed by the hypermethylation of their promoter regions [10]. Treatment of cells with 5-aza-2’-deoxycytidine, a methyl transferase inhibitor, can alleviate this suppression. The expression of miR-9 in ARPE-19 cells increased approximately threefold in response to the treatment (Figure 5). *TGM2*, a gene known to be regulated by hypermethylation [28], was also induced as expected.

**Figure 6:**
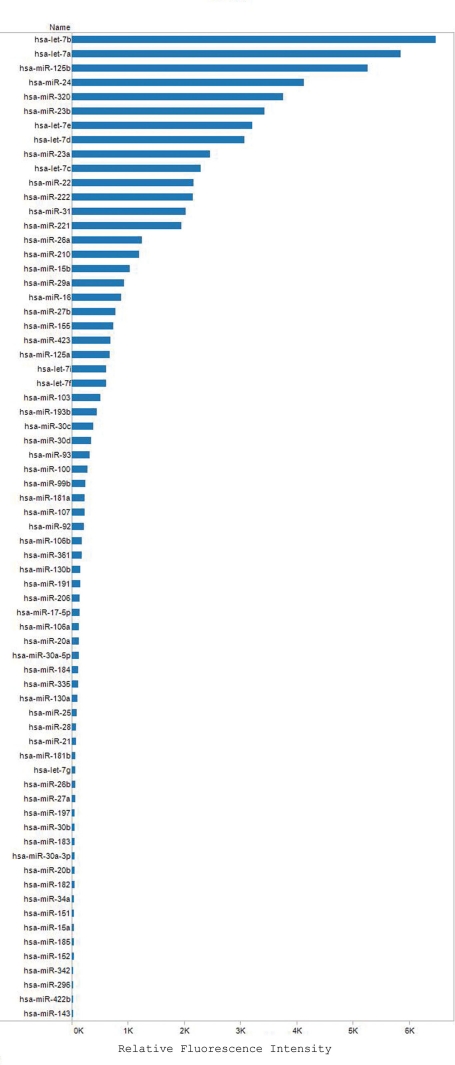
Microarray hybridization analysis of miRNAs normally expressed in ARPE-19 cells. The data shown is representative of hybridization analyses performed using two RNA samples isolated from untreated ARPE-19 cells. The microarrays contained duplicate spots for each miRNA probe. The average fluorescence intensities are shown; the view is filtered by name, which has multiple members selected.

We next used microarray hybridization analysis to obtain a broad view of the microRNA species normally expressed in ARPE-19 cells. Small RNA preparation, which was enriched from total RNA isolated from untreated ARPE-19 cells, was employed for hybridization analysis on a microarray containing duplicate spots of each miRNA probe. The microarray analysis, when repeated using a second RNA sample, yielded similar results. The microarray data are provided at GEO (GSE23107). The relative expression levels of 62 miRNA (out of 366 human miRNAs tested) expressed substantially in these cells are shown in Figure 6. The most abundant miRNAs expressed in ARPE-19 cells were let-7b, let-7a, miR-125b, miR-24, miR-320, miR-23b, let-7e, and let-7d. The microarray analysis failed to detect the miR-9 expression in ARPE-19 cells. This could be due to the limited sensitivity of microarray analysis in comparison to real-time RT–PCR, where the target is detected after multiple cycles of amplification.

**Figure 7:**
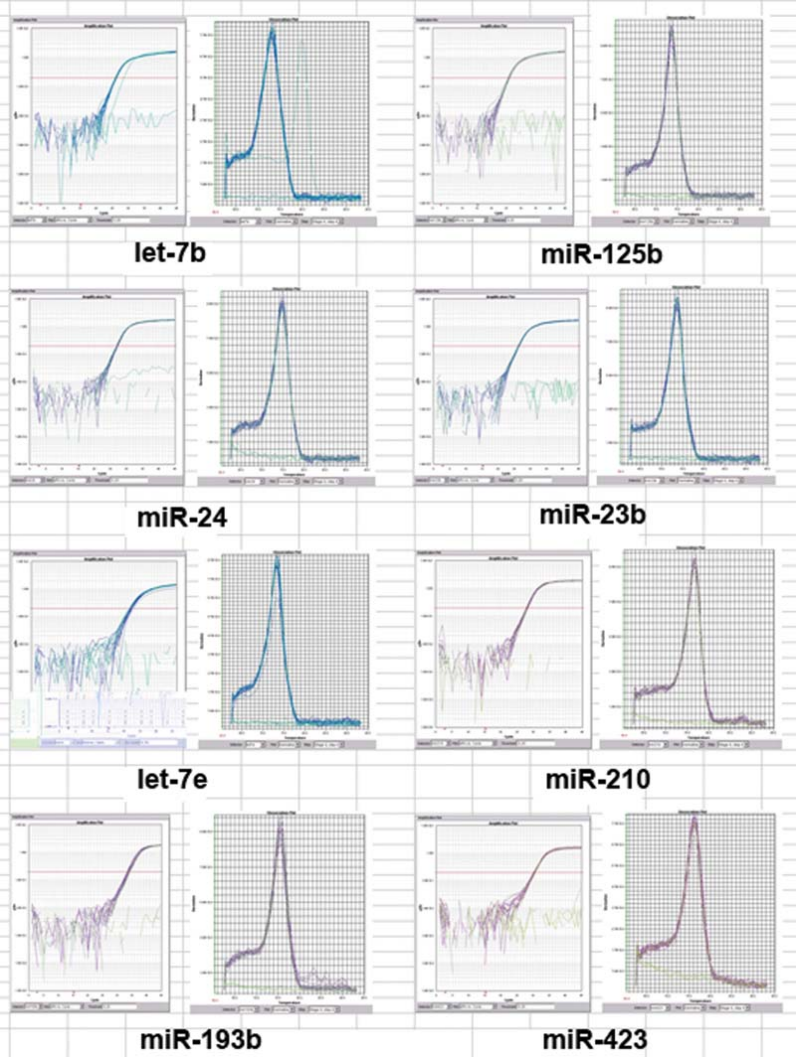
Real-time RT–PCR validation of selected miRNAs. Amplification plot (left panel) and dissociation plot (right panel) for the indicated miRNA are shown.

**Figure 8:**
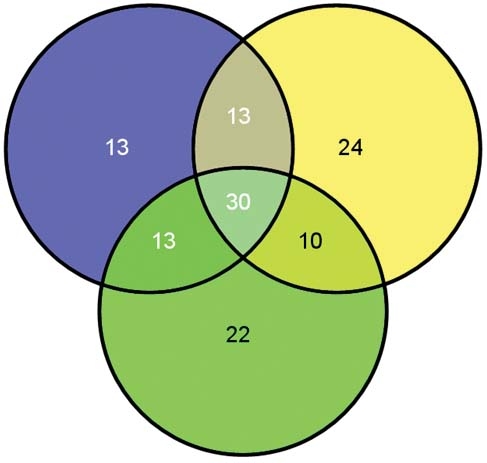
The miRNAs expressed in ARPE-19 cells are compared to those found in mouse retina, as reported in two separate studies. Blue circle: ARPE-19 cells. Yellow circle: mouse retina, Xu et al. [37]. Green circle: mouse retina (regulated by ischemia), Shen et al. [55].

The microarray results were validated by analyzing the expression of selected miRNAs using real-time PCR analysis. Let-7b, miR-125b, miR-24, miR-23b, and let-7e represented the most abundant ones, while miR-210, miR-193b and miR-423 represented the less abundant ones. The amplification and dissociation plots (Figure 7) clearly indicate that all the miRNAs tested are present in the RNA preparations obtained from the ARPE-19 cells. Several miRNAs expressed in ARPE-19 cells are also reported or predicted to be neural retina-specific (Figure 8).

**Figure 9:**
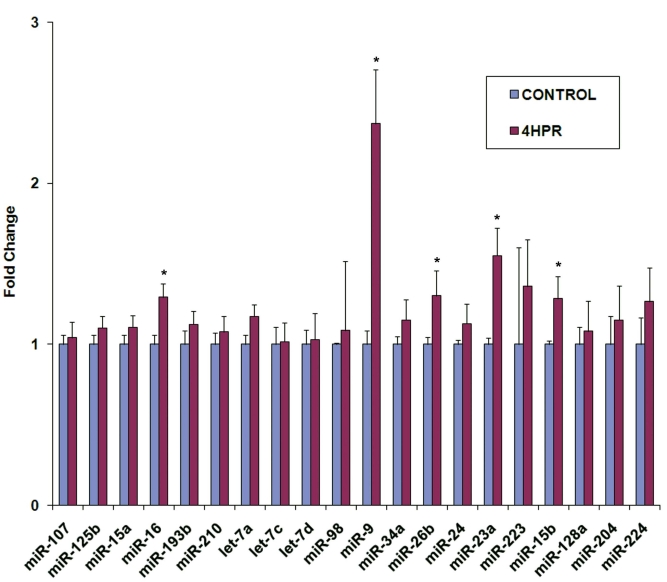
Effect of 4HPR on miRNA expression in ARPE-19 cells. The cells were treated with 10 μM 4HPR for 24 h, and the expression of miRNAs was analyzed using real-time RT–PCR. The relative expression levels for each miRNA in control and treated samples were estimated and used to calculate the fold change. *p<0.05 compared to control, n=4.

We analyzed the response of selected miRNAs in ARPE-19 cells to 4HPR treatment using real-time RT–PCR analysis (Figure 9). Most of the miRNAs tested were selected from microarray results shown in Figure 6 to represent both the most abundant as well as the less abundant miRNAs. The miR-98, miR-223, miR-15b, and miR-128a were included to represent the ones not detected by microarray analysis. We also tested miR-204 and miR-224 because they are reported to be expressed in mouse RPE [29,30]. Although all the tested miRNAs, including miR-204 and miR-224, were found to be expressed in ARPE-19 cells, the majority did not respond to 4HPR treatment. Increases in the expression of miR-16, miR-26b, miR-23a, and miR-15b were observed following 4HPR treatment; however, these increases were modest when compared to the approximately twofold increase observed for miR-9. The 5′-flanking regions (~1 kb) of genes generating miR-16, miR-26b, miR-23a, miR-15b, miR-223, and let-7a were analyzed for the presence of consensus binding sites for CEBP-α and CEBP-β. The genes for miR-16, miR-15b, and miR-223 as well as one of the three genes encoding let-7a were found to contain potential binding sites for these transcription factors (data not shown).

## Discussion

Although microRNA expression and function in the mammalian eye has been investigated [29,31-37], information about miRNA expression and function in the RPE is limited. An exception is the reported detection of miR-204 and miR-224 in mouse RPE using in situ hybridization [29,30]. Here we demonstrate that miR-9 is expressed in ARPE-19 cells, and that its expression is increased when these cells are exposed to 4HPR. The increase in miR-9 expression by 4HPR was associated with apoptosis, as indicated by mono- and oligonucleosomes formation, and with large increases in *HMOX1* and *GADD153* expression. The 4HPR-induced apoptosis and increases in *GADD153* and *HMOX1* expression in ARPE-19 cells are preceded by an increase in reactive oxygen species generation in ARPE-19 cells [26]. Taken together, these facts are consistent with a model of miR-9 activation due to reactive oxygen species generated from 4HPR treatment. Indeed, increase of miR-9 expression following oxidative stress induced by metal sulfates has been reported [18]. However, our results eliminate oxidative stress as a direct regulator of the miR-9 expression in ARPE-19 cells. Menadione and arsenite, agents well known to cause oxidative stress, failed to increase the miR-9 expression in these cells. In contrast, the expression of *HMOX1*, a marker for oxidative stress, was highly increased in cells treated with these agents, as expected. 4HPR is a derivative of all-trans-retinoic acid, which has been shown to be a regulator of miR-9 expression [17]. All-trans-retinoic acid is also known to regulate gene expression through its effects upon the expression of CEBP transcription factors [38,39]. Our sequence analysis indicated that putative CEBP binding sites are present on potential promoter regions of all three genes encoding miR-9 precursors (*MIR9–1*, *MIR9–2*, and *MIR9–3*). Our observation that the expression of genes encoding CEBP-α and CEBP-β increased in ARPE-19 cells in response to 4HPR treatment suggests that these transcription factors could be involved in the regulation of miR-9 expression. The 4HPR treatment also significantly increased the expression of miR-15b, miR-16, miR-23a, and miR-26b. However, putative binding sites for CEBP-α and CEBP-β were absent in the promoter regions of the genes encoding miR-23a and miR-26b. Thus, factors other than CEBP-α and CEBP-β are indicated in the regulation of expression of these miRNAs by 4HPR.

Expression of miR-9 is also known to be regulated by hypermethylation of the promoter regions of genes encoding this microRNA. This epigenetic repression of miR-9 expression has been reported to be a contributing factor to breast and colorectal cancer development [10-12]. Our results show that the miR-9 expression in ARPE-19 cells is regulated by this mechanism. Treatment of the ARPE-19 cells with 5-aza-2’-deoxycytidine to block DNA methylation resulted in increased expression of both miR-9 and *TGM2*, a gene known to be regulated by hypermethylation [28]. The potential role of epigenetic regulation of miR-9 or other microRNAs in RPE pathophysiology is not yet known, although it has been implicated in cancer and aging [40,41].

The ability of miR-9 to target many genes for translational repression makes it a potentially effective regulator of cell growth, differentiation, neurogenesis, immunity and cancer. There appears to be a large number of potential targets for this microRNA; 936 conserved targets of miR-9 are reported in TargetScan release 5.2. However, only a few of them have been verified experimentally. MiR-9 is reported to control the secretory response of insulin-producing cells by diminishing the expression of the *ONECUT2* transcription factor, a regulator of Granuphilin expression [3]. The transcription factor *REST*, a silencer of neuronal gene expression, is also a target of miR-9 whose expression is downregulated in Huntington’s disease [4]. MiR-9 is reported to accelerate neural stem cell differentiation by suppressing the expression of the nuclear receptor *TLX* [5]. Another target for miR-9 is BACE1/beta-secretase, whose activity is elevated in brains affected by Alzheimer disease [14]. The transcription factor *NF-KappaB1* is reported to be targeted by this microRNA during growth of ovarian cancer cells and in monocytes and neutrophils exposed to pro-inflammatory agents [6,7]. Several components of the Fgf signaling pathway are targeted by miR-9 during late embryonic development in zebrafish [8]. Translational repression by miR-9 is also reported for *E-cadherin* in the SK-Hep-1 hepatoma cell line [42], *Foxg1* in developing mouse brains [43] and the BK channel splice variant in rat striatal neurons during alcohol adaptation [19]. *ONECUT2* appears to be an interesting target since it has been reported that this transcription factor regulates the expression of *MITF*, a gene important for RPE physiology [44]. *NF-KappaB1* could also be an important target during the response of RPE to pro-inflammatory agents or oxidative stress.

We demonstrated using microarray analysis that a large number of miRNAs are normally expressed in the RPE cell line, ARPE-19. RT–PCR analysis was employed to verify the expression of several miRNAs. The most abundant miRNAs that were detected in ARPE-19 cells were let-7b, let-7a, miR-125b, miR-24, miR-320, miR-23b, let-7e, and let-7d. The let-7 family of microRNAs are important regulators of differentiation and development, and their deregulation is often associated with cancer [45,46]. MiR-125b is a novel regulator of the tumor suppressor p53 [47] as well as many genes involved in neuronal differentiation [48]. MiR-24 has been shown to regulate several cell cycle genes, the activin type 1 receptor ALK4 and dihydrofolate reductase [49-51]. MiR-320 regulates cardiac ischemia/reperfusion injury and cell proliferation by targeting heat-shock protein 20 and transferrin receptor 1, respectively [52,53]. MiR-23b is reported to regulate cell differentiation by targeting Smad proteins [54]. The potential role of these microRNAs in regulating RPE function remains to be elucidated. It should be noted that microarray analysis did not detect miR-9 expression in ARPE-19 cells. The sensitivity of microarray analysis is highly limited when compared to real-time RT–PCR, the method we first employed to detect it.

Since ARPE-19 cells are a widely employed and useful experimental model, it is sensible to determine how well the population of miRNAs corresponds to what is observed in retinal tissue. Two groups of investigators have studied miRNA expression in mouse retinas, using both array hybridization techniques. Among the 69 miRNAs identified in this study, the 77 reported by Xu et al. [37] and 75 reported by Shen et al. [55], 30 are common to all three sets. The two studies based upon extraction of miRNA from retinal tissue have a total of 40 miRNAs in common. Interestingly, the ARPE-19 cells have a similar degree of commonality with the 2 data sets from retinas, having 43 in common with each.

The hybridization studies above have been complemented by an informatics approach. Working from a collection of retinal genes, Arora et al. [33] produced a list of miRNAs predicted to target the 3′-untranslated regions of their mRNA sequences. The authors then demonstrated the expression of a selection of the miRNAs using PCR. Several of the verified miRNAs are present in all three hybridization data sets (miR-29, miR-107, let-7d, miR-23a, and miR-143); three others (miR-124, miR-106, and miR-143) are present in at least one of the hybridization data sets. MiR-135 and miR-200, although validated, were not found in any of the hybridization data sets. However, this validation was performed using PCR, and like miR-9 in the present study, the expression of miR-135 and miR-200 may be undetectable by hybridization. Only 10 of the miRNAs found collectively in the three hybridization studies were not among those predicted. Interestingly, 5 of these (miR-184, miR-210, miR-31, miR-335, and miR-92) were deemed to be “retina specific” in expression, according to the tissue survey conducted by Xu et al. [37]. Thus, it appears that the predictive value of the informatics approach is fruitful.

In summary, we have shown that miR-9 is expressed in the RPE cell line known as ARPE-19 and that its expression increases during 4HPR-induced apoptosis. The 4HPR-induced miR-9 expression was associated with parallel increases in the expression of *CEBPA* and *CEBPB* transcription factor genes. Potential binding sites for these transcription factors were present in the putative promoter regions of all three genes encoding miR-9. The miR-9 expression in ARPE-9 cells was also increased in response to treatment with 5-aza-2’-deoxycytidine, a methyl transferase inhibitor, thus indicating that the gene(s) encoding this microRNA could be epigenetically regulated via hypermethylation of their promoter regions. Microarray analysis showed that a large number of miRNAs are normally expressed in ARPE-19 cells, the most abundant ones being let-7b, let-7a, miR-125b, miR-24, miR-23b, let-7e, and let-7d. The miR-9 and other microRNAs could play an important role in maintaining RPE cell function.
